# The Erie County Medical Society—The Annual Meeting

**Published:** 1882-01

**Authors:** 


### The Erie County Medical Society.

### THE ANNUAL MEETING.

On Tuesday, January 10th, the annual meeting of the Medical Society of the county of Erie will be held at the Buffalo Medical College. The business, coming before these annual gatherings of the profession, possesses little of general interest, but the meetings being required by statutory provision, bring together members from city and towns alike, and through mutual interchange of thought and feeling lead to a better acquaintance and to a wider sympathy and more earnest interest in the general interests of the profession.

The most important matter to be considered at this meeting is the action of the Board of Censors in relation to the new medical law. At the last annual meeting authority was given to institute legal proceedings to test the validity of the charter of a medical school, located in this city, which was issuing diplomas without due warrant of law. We have published the result of the trial, as given in the able decision of the Hon. Geo. Barker, Judge of the Supreme Court, and published in full in the September number of this journal. The Censors, successful in their first step against a dangerous source of bogus diplomas, have, as far as we are informed, ceased from their labors, to await, we presume, further instructions from the Society. But the inquiry is a pertinent one; why wait for the annual meeting? Is the medical law, from which so much was expected, so loosely drawn up that imposters and quacks can slip through its meshes with impunity and defy both law and decency by continuing their nefarious traffic with human life? Is there no protection for legitimate medicine except that which proceeds from its own inherent merits, and the recognition of its earnest labors, and its sacrifices in behalf of the maimed and the suffering from the enlightened and intelligent sentiment of the public? We confess to a feeling of distrust for all and every movement inaugurated for the special purposes for which past legislation has been enacted, if this effort fails to accomplish anything, either to protect the public or to benefit the profession. To the Board of Censors we look for an explanation of their long silence, which we do not wish to construe as due to apathy towards the great interests involved in the vital questions intrusted to their charge, and we hope their report will present the entire subject, and their action in the premises in a satisfactory light before the Society. Until that time we refrain from further criticism as to the law and its operations.

